# Detection of tick-borne pathogens in questing *Ixodes ricinus* in the French Pyrenees and first identification of *Rickettsia monacensis* in France

**DOI:** 10.1051/parasite/2019019

**Published:** 2019-04-03

**Authors:** Toufic Akl, Gilles Bourgoin, Marie-Line Souq, Joël Appolinaire, Marie-Thérèse Poirel, Philippe Gibert, Georges Abi Rizk, Mathieu Garel, Lionel Zenner

**Affiliations:** 1 Université Libanaise, Faculté d’Agronomie et de Médecine Vétérinaire, Département de Médecine Vétérinaire 6573 Beyrouth Liban; 2 Université de Lyon, VetAgro Sup – Campus Vétérinaire de Lyon, Laboratoire de Parasitologie Vétérinaire 1 avenue Bourgelat, BP 83 69280 Marcy l’Etoile France; 3 Université de Lyon, Université Lyon 1, CNRS, UMR 5558, Laboratoire de Biométrie et Biologie Évolutive 69622 Villeurbanne France; 4 Office National de la Chasse et de la Faune Sauvage, Unité Ongulés Sauvages 5 allée de Bethléem, 9 Z.I. Mayencin 38610 Gières France

**Keywords:** *Ixodes ricinus*, Tick-borne infections, Public health, Pyrenean chamois, *Rickettsia monacensis*

## Abstract

Ticks are important vectors of several human and animal pathogens. In this study, we estimated the prevalence of important tick-borne infections in questing ticks from an area in Southwestern France (Hautes-Pyrénées) inhabited by Pyrenean chamois (*Rupicapra pyrenaica pyrenaica*) experiencing high tick burden. We examined adult and nymph ticks collected by the flag dragging method from 8 to 15 sites in the Pic de Bazès during the years 2009, 2011, 2013 and 2015. PCR assays were conducted on selected ticks for the detection of *Borrelia burgdorferi* s.l.*, Babesia* spp., *Rickettsia* spp., spotted fever group (SFG) *Rickettsia* and *Anaplasma phagocytophilum*. Randomly selected positive samples were submitted for sequence analysis. A total of 1971 questing ticks were collected including 95 males, 101 females and 1775 nymphs. All collected ticks were identified as *Ixodes ricinus.* Among them, 696 ticks were selected for pathogen detection and overall prevalence was 8.4% for *B. burgdorferi* s.l.; 0.4% for *Babesia* spp.; 6.1% for *A. phagocytophilum*; 17.6% for *Rickettsia* spp.; and 8.1% for SFG *Rickettsia*. Among the sequenced pathogens, we detected in this population of ticks the presence of *Babesia* sp. EU1 and *Rickettsia helvetica*, as well as *Rickettsia monacensis* for the first time in France. The detection of these pathogens in the Pic de Bazès highlights the potential infection risks for visitors to this area and the Pyrenean chamois population.

## Abstract (fr)

Les tiques sont des vecteurs importants de plusieurs maladies animales et humaines. Dans cette étude, nous avons estimé la prévalence de maladies vectorisées chez les tiques errantes dans une région du sud-ouest de la France (Hautes-Pyrénées) occupée par des chamois des Pyrénées (*Rupicapra pyrenaica pyrenaica*) fortement infestés par les tiques. Nous avons utilisé des tiques adultes et des larves collectées par la technique du drapeau sur 8–15 sites du Pic de Bazès au cours des années 2009, 2011, 2013 et 2015. Des analyses PCR ont été menées sur des tiques sélectionnées pour la détection de *Borrelia burgdorferi* s.l., *Babesia* spp., *Rickettsia* spp., *Rickettsia* du groupe de la fièvre pourprée (GFP) et *Anaplasma phagocytophilum*. Des échantillons positifs sélectionnés au hasard ont été soumis à une analyse de séquences. Un total de 1971 tiques a été collecté dont 95 mâles, 101 femelles et 1775 larves. Toutes les tiques recueillies ont été identifiées comme étant *Ixodes ricinus*. Parmi celles-ci, 696 tiques ont été sélectionnées pour la détection des agents pathogènes et la prévalence globale était de 8,4 % pour *B. burgdorferi* s.l., 0,4 % pour *Babesia* spp., 6,1 % pour *A. phagocytophilum*, 17,6 % pour *Rickettsia* spp. et 8,1 % pour *Rickettsia* GFP. Parmi les agents pathogènes séquencés, nous avons détecté dans cette population de tiques la présence de *Babesia* sp. EU1, *Rickettsia helvetica* et, pour la première fois en France, *Rickettsia monacensis*. La détection de ces agents pathogènes dans le Pic de Bazès souligne les risques potentiels d’infection pour les visiteurs de cette région et pour la population de chamois des Pyrénées.

## Introduction

Ticks are a major threat to human and animal health as they are one of the most important arthropod vectors of pathogens for both humans and wild and domestic animals. These pathogens include *Borrelia burgdorferi* s.l., *Anaplasma phagocytophilum*, *Rickettsia* spp., and *Babesia* spp. [19, 33, 47].

Climate change has a major impact on tick population distribution and dynamics, notably with the development of these arthropods in increasingly high densities at relatively high altitudes [12], as well as a shift in patterns of seasonal activity [14, 32]. This could widen the interface of these vectors to include hikers, domestic animals feeding on high altitude pastures, and wild animals inhabiting these high mountains, such as wild ungulates. These changes in tick dynamics are of serious concern as ticks are pathogen vectors and these changing dynamics could lead to increased transmission of tick-borne diseases [14].

The role of wild ungulates as reservoir hosts for *Anaplasma phagocytophilum*, spotted fever group (SFG) *Rickettsia* and *Borrelia burgdorferi* s.l. (*sensu lato*) is not completely understood [24], since these pathogens are not frequently associated with clinical disease. However, wild ungulates are known to play a role in the maintenance of these bacteria in nature [7, 31]. Clinical babesiosis has not often been described in wild ungulates, with the first case being detected in alpine chamois (*Rupicapra rupicapra rupicapra*) from Switzerland in 1964 [5]. This chamois showed extreme anemia, icterus, hepatomegaly and splenomegaly, and *Babesia bovis* was identified by microscopic examination of a blood smear. Since then, babesiosis was repeatedly detected in chamois [17, 18, 29]. In 2005, a chamois from the Swiss Alps with symptoms of piroplasmosis showed erythrocytic inclusions that were later confirmed by PCR and sequencing as *Babesia capreoli*, marking the first reported PCR identification of this parasite in clinically ill chamois [43].

Since 2002, we have observed relatively low kid spring survival (0.52 ± 0.04) with large year to year variations (from 0 to 0.75, CV = 42%) in a population of Pyrenean chamois (*Rupicapra pyrenaica pyrenaica*) in the French Pyrenees (Pic de Bazès, Hautes-Pyrénées) [11, 41, 42]. Among the few corpses recovered, some had a high tick burden (>100 ticks), often associated with anemia, hemorrhage and splenomegaly, but the cause of death remained undetermined despite several laboratory investigations. Six apparently healthy individuals out of 10 trapped in 2008 had a positive PCR result for *B. capreoli* and *B. venatorum* (formerly known as *Babesia* sp. EU1), and 2 out of 5 (two dead and three shot) were positive by PCR for *B. divergens* in 2010 [13].

On the basis of these findings, a survey of the population of ticks in the Pic de Bazès was initiated in 2009. Based on the ticks collected in this survey, we investigated the critical pathogens harboured by this tick population.

## Materials and methods

### Ticks collection and identification

In this study, we used samples collected every other year from 2009 to 2015 (i.e., 2009, 2011, 2013, 2015) in the Pic de Bazès, in the foothills of the French Western Pyrenees (43.00°N, 0.23°W), in the department of Hautes-Pyrénées in southwestern France.

The study area encompasses 400 ha between 1000 and 1800 m a.s.l., is mostly covered by alpine grass (*Festuca eskia*), rocks and forest (beech *Fagus sylvatica* and firs *Abies* sp.), and is inhabited by a population of 100–130 Pyrenean chamois [22]. During the study period, the average (±*SD*) annual minimum and maximum daily temperatures were 5.8 ± 5.7 °C and 15.1 ± 7.3 °C, respectively (at 910 m a.s.l.). Total annual precipitations reached 1297 ± 271 mm.

Ticks were collected from 15 different sites in the study area until June 2013 and from the eight most infested sites afterwards. Ticks were collected by dragging a white flag of 1 m^2^ on vegetation along a 30 m line in each site. Sampling was repeated each month, from spring to fall. Collected ticks were placed in glass tubes with 70% ethanol or with a small piece of humid cotton. Due to the small size of larvae, they were not collected from the dragging flag.

Collected tick samples were transported to the laboratory and then identified using a binocular microscope. Species, stage and sex (for adults) were identified using morphological criteria following standard taxonomic keys [9, 37]. Ticks were stored individually in 1.5 mL plastic tubes with 70% ethanol and stored at −20 °C, or directly in a dry tube at −80 °C.

### DNA extraction

As only a few adult specimens were collected per year, we extracted the DNA from all of them except in 2013, since they were abundant that year. We completed this dataset by randomly selecting adult ticks collected in 2013 (from the different sites and sampling periods), and nymphs (throughout the different years, sampling sites and periods). Prior to DNA extraction, ticks were individually washed twice for 10 min in 800 μL of 70% ethanol then in sterile phosphate buffered saline (PBS), after which ticks were transferred into new 1.5 mL tubes and washed a final time with PBS. The tubes were vigorously vortexed after each bath and finally, PBS was removed to allow ticks to dry. Each tick was incised into small pieces while in the tube with a disposable no. 11 scalpel blade. DNA was then extracted from each tick using NucleoSpin^®^ Tissue (Macherey-Nagel, Düren, Germany) for adults, and NucleoSpin^®^ Tissue XS (Macherey-Nagel, Düren, Germany) for nymphs. At the final extraction step, DNA was eluted in 100 μL of kit solution for adult ticks, and in 40 μL for nymphs. Extracted DNA was stored at −20 °C prior to molecular analyses.

### Molecular analyses

The quality of the extracted DNA was verified by PCR amplification of a 320 bp region of the mitochondrial 16S rDNA specific to ticks using the primers TQ 16S+1F and TQ 16S−2R, as described in Table 1 [2]. A positive control consisting of *Ixodes ricinus* DNA and a negative control consisting of PCR product mix with no DNA were included. PCR products were then stained with bromophenol blue and examined by gel electrophoresis (1.5% agarose gel, Standard Agarose, Eurobio, France), and detected using ultraviolet light (Kodak EDAS 290, New York, USA).

**Table 1:** Primers used in the PCR assays conducted in this study with their respective references.

Organism	Targeted gene (product size)	Name and primer sequences (5′ – 3′)	PCR conditions	Ref.
Tick	Mitochondrial 16S rDNA (320 bp)	TQ 16S+1F:	Denaturation	[2]
		5′-CTGCTCAATGATTTTTTAAATTGCTGTGG-3′	94 °C 5 min	
		TQ 16S−2R:	Hybridization	
		5′-ACGCTGTTATCCCTAGAG-3′	10 cycles: 92 °C 1 min; 48 °C 1 min; 72 °C 1 min 30 s	
			32 cycles: 92 °C 1 min; 54 °C 1 min; 72 °C 1 min 30 s	
			Extension	
			72 °C 5 min	
Anaplasmataceae	16S rRNA (345 bp)	EHR 16SD:	Denaturation	[36]
		5′-GGTACCYACAGAAGAAGTCC-3′	94 °C 5 min	
		EHR 16SR:	Hybridization	
		5′-TAGCACTCATCGTTTACAGC-3′	34 cycles: 94 °C 40 s; 50 °C 40 s; 72 °C 1 min	
			Extension	
			72 °C 10 min	
*Anaplasma phagocytophilum*	16S rDNA (932 bp)	ge3a:	Denaturation	[28]
		5′-CACATGCAAGTCGAACGGATTATTC-3′	95 °C 5 min	
		ge10r:	Hybridization	
		5′-TTCCGTTAAGAAGGATCTAATCTCC-3′	40 cycles: 94 °C 30 s; 55 °C 30 s; 72 °C 1 min	
			Extension	
			72 °C 5 min	
	16S rDNA (546 bp)	ge9f:	Denaturation	
		5′-AACGGATTATTCTTTATAGCTTGCT-3′	95 °C 5 min	
		ge2r:	Hybridization	
		5′-GGCAGTATTAAAAGCAGCTCCAGG-3′	30 cycles: 94 °C 30 s; 55 °C 30 s; 72 °C 1 min	
			Extension	
			72 °C 5 min	
*Borrelia burgdorferi* s.l.	16S rRNA (357 bp)	16S LDF:	Denaturation	[27]
		5′-ATGCACACTTGGTGTTAACTA-3′	95 °C 5 min	
		16S LDR:	Hybridization	
		5′-GACTTATCACCGGCAGTCTTA-3′	35 cycles: 95 °C 1 min; 53 °C 1 min; 72 °C 1 min	
			Extension	
			72 °C 10 min	
*Babesia/Theileria* spp.	18S rRNA (359 bp)	BAB GF2:	Denaturation	[4]
		5′-GYYTTGTAATTGGAATGATGG-3′	94 °C 5 min	
		BAB GR2:	Hybridization	
		5′-CCAAAGACTTTGATTTCTCTC-3′	35 cycles: 94 °C 1 min; 60 °C 1 min; 72 °C 1 min	
			Extension	
			72 °C 10 min	
*Rickettsia* spp.	gltA (citrate synthase) (381 bp)	RpCS.877p:	Denaturation	[39]
		5′-GGGGGCCTGCTCACGGCGG-3′	95 °C 5 min	
		RpCS.1258n:	Hybridization	
		5′-ATTGCAAAAAGTACAGTGAACA-3′	6 cycles: 94 °C 1 min; 60 °C 1 min; 72 °C 1 min	
			30 cycles: 94 °C 1 min; 53 °C 1 min; 72 °C 1 min	
			Extension	
			72 °C 10 min	
SFG *Rickettsia*	ompA (532 bp)	Rr190.70p:	Denaturation	[39]
		5′-ATGGCGAATATTTCTCCAAAA-3′	95 °C 5 min	
		Rr190.602n:	Hybridization	
		5′-AGTGCAGCATTCGCTCCCCCT-3′	6 cycles : 94 °C 1 min; 60 °C 1 min; 72 °C 1 min	
			30 cycles : 94 °C 1 min; 53 °C 1 min; 72 °C 1 min	
			Extension	
			72 °C 10 min	

All positive DNA samples were screened for the presence of DNA of *Borrelia burgdorferi* s.l., Anaplasmataceae, *Babesia/Theileria* spp. and *Rickettsia* spp. Independent PCR assays were conducted using the primers and conditions presented in Table 1. Nested PCR assay using ge3a/ge10r as the first primer couple and ge9f/ge2r as the second, amplifying a part of the 16S rDNA specific to *Anaplasma phagocytophilum* was conducted only on positive samples to Anaplasmataceae, except for the samples collected in 2009, which were previously tested directly with the *A. phagocytophilum* nested PCR. Similarly, for SFG *Rickettsia*, only samples that were positive for *Rickettsia* spp. were tested. PCR assays were further analyzed using the primer couple Rr190.70p/Rr190.602n, amplifying the ompA gene specific for SFG *Rickettsia*. Positive and negative controls were included in each run. All amplified products were examined by gel electrophoresis as described above.

### Sequence analysis

A representative number of positive samples for each tested pathogen showing clear positive strands on gel electrophoresis were randomly selected for sequence analysis. Samples were sent to Biofidal Laboratory (Villeurbanne, France) for sequencing in both directions using the same primers as the ones employed in the PCR assays. We used CLC Main Workbench 8 (Qiagen, Hilden, Germany) to analyze the quality of the sequences and create consensus sequences. This consensus was compared with sequences available from the GenBank^®^ database with the BLAST tool of the CLC Main Workbench.

### Statistical analyses

Fisher’s exact tests were used to study the effects of tick stage (i.e., adult or nymph) and year of collection on the prevalence of pathogens. Statistical analyses were conducted with the software STATISTICA 10^®^ (Stat Soft Inc., 2011). A significance threshold level of 0.05 was used.

## Results

### Ticks collected

A total of 1971 questing ticks were collected during the study period including 95 males, 101 females and 1775 nymphs. All ticks were identified as *Ixodes ricinus.*

DNA extraction and PCR assays for the detection of vector-borne pathogens were conducted on 79 males, 86 females and 531 nymphs (i.e., 83.2%, 85.1% and 29.9% of the total number of collected males, females and nymphs, respectively) sampled from each site and year (see Table 2 for details on the number of ticks per year).

**Table 2:** The results of PCR assays for the different tested pathogens with the corresponding percentages.

Year	*Borrelia burgdorferi* s.l.		*Babesia*/*Theileria* spp.		*Rickettsia* spp.		SFG *Rickettsia*		Anaplasmataceae		*A. phagocytophilum*	
	positive/tested	%	positive/tested	%	positive/tested	%	positive/tested	%	positive/tested	%	positive/tested	%
2009												
Male	0/13	0	0/13	0	0/13	0	–	–	–	–	0/13	0
Female	0/9	0	0/9	0	1/9	11.1	0/1	0	–	–	1/9	11.1
Nymph	5/133	3.7	2/133	1.5	0/133	0	–	–	–	–	6/133	5
Total	5/164	3.0	2/164	1.2	1/164	0.6	0/1	0	–	–	8/164	4.8
2011												
Male	2/21	9.5	0/21	0	11/21	52.3	1/11	9.0	1/21	4.7	0/1	0
Female	3/26	11.5	0/26	0	14/26	53.8	2/14	14.2	25/26	96.1	0/25	0
Nymph	0/137	0	0/137	0	46/137	33.5	0/46	0	15/137	10.9	3/15	20
Total	5/184	2.7	0/184	0	71/184	38.5	3/71	4.2	41/184	22.2	3/41	7.3
2013												
Male	2/31	6.4	0/31	0	8/31	25.8	0/8	0	2/31	6.4	1/2	50
Female	0/29	0	0/29	0	4/29	13.7	0/4	0	7/29	24.1	0/7	0
Nymph	47/170	27.6	1/170	0.5	27/170	15.8	3/27	11.1	37/170	21.7	4/37	11
Total	49/230	21.3	1/230	0.4	39/230	16.9	3/39	7.6	46/230	20.0	5/46	10.8
2015												
Male	0/14	0	0/14	0	1/14	7.1	1/1	100	1/14	7.1	0/1	0
Female	0/22	0	0/22	0	9/22	40.9	2/9	22.2	18/22	81.8	1/18	5.5
Nymph	0/91	0	0/91	0	2/91	2.2	1/2	50.0	1/91	1.1	0/1	0
Total	0/127	0	0/127	0	12/127	9.4	4/12	33.3	20/127	15.7	1/20	5.0
**Total**	**59/696**	**8.4**	**3/696**	**0.4**	**123/696**	**17.6**	**10/123**	**8.1**	**107/541**	**19.7**	**16/262**	**6.1**

### Detection of *Borrelia burgdorferi* s.l.

DNA of *Borrelia burgdorferi* s.l. was found in 8.4% of tested ticks (59/696) with significant strong annual (0–21.3%) and site (0–29.4%) variations (Table 2). Prevalence of infection in questing ticks in 2013 was 21.3%, making it significantly higher than all the other years (*p* < 0.05). Questing nymphs collected over the four years were found to have a significantly higher infection rate (*p* < 0.05) than adult ticks (9.7% vs. 4.2%; *p* < 0.05), except in 2011 (0% vs. 10.7%).

Fifteen of the 59 *Borrelia burgdorferi* s.l. positive samples were further analyzed by sequencing. The five samples from 2009 that were sequenced produced highly similar consensus sequences of 351 nucleotides that revealed 100% similarity with sequences of the 16S gene of *Borrelia afzelii* isolated from ticks and humans (GenBank accession numbers EF541175 and CP002933, respectively). The remaining samples (i.e., 5 from 2011 and 5 from 2013; 258–354 bp) showed 99–100% similarity with sequences of different genospecies of *B. burgdorferi* s.l. complex, such as cultured *B. burgdorferi* s.s. and *B. garinii* isolated from *Ixodes* spp. ticks (GenBank accession numbers CP031412 and KY346890, respectively).

### Detection of *Babesia/Theileria* spp.

Molecular analyses revealed the presence of *Babesia/Theileria* spp. DNA in three of the 696 tested samples (prevalence of 0.4%). Two of these positive samples were collected in 2009 and the third one was detected in a sample from 2013 (Table 2).

All three positive samples were submitted for sequence analysis. The two samples collected in 2009 showed the closest similarity (99.1%) to a sequence of the 18S gene of *Babesia* sp. EU1 isolated from roe deer in France (GenBank accession number HQ830266). The positive sample collected in 2013 (722 bp) showed 99.7% similarity to a sequence of *Babesia* sp. EU1 isolated from *I. ricinus* ticks in the Czech Republic (GenBank accession number KX857480).

### Detection of *Rickettsia* spp.

The overall prevalence of *Rickettsia* spp. was found to be 17.6% (123/696), with statistically significant annual (0.6–38.5%) and site (9.6–50%) variations (Table 2). Adult ticks had a significantly higher infection prevalence than nymphs (29.0% (48/165) vs. 14.1% (75/531); *p* < 0.05).

An additional PCR assay was conducted on all positive samples with a probe amplifying the ompA gene specific to SFG *Rickettsia* which revealed 10 positive samples (8.1%), with the highest prevalence in 2015 (4/10). Tick stage and site of collection showed no significant effect on prevalence.

Five of the 10 positive SFG *Rickettsia* samples were submitted for sequence analysis. The consensus sequences (510–530 bp) showed 100% similarity to the ompA gene sequence of *R. monacensis* isolated from *I. ricinus* larvae in Italy (GenBank accession number KY319223). Two samples positive with the gltA probe, but negative with ompA, were submitted for sequence analysis with the gltA primer. Consensus sequences (384–385 bp) showed 100% similarity to *R. helvetica* isolated from *I. ricinus* ticks infesting dogs in central Italy (GenBank accession number KY231198).

### Detection of Anaplasmataceae and *Anaplasma phagocytophilum*

PCR assay conducted for the detection of the DNA of Anaplasmataceae on samples collected in 2011, 2013 and 2015 revealed a prevalence of 19.7% (107/541; Table 2). A nested PCR that identifies *Anaplasma phagocytophilum* was conducted on all positive samples, in addition to all the samples collected in 2009. Overall prevalence of *A. phagocytophilum* was found to be 6.1% (16/262). No significant effect of tick stage and year of collection on the prevalence of *A. phagocytophilum* were detected.

Three positive samples from 2011 and one from 2015 out of the 15 positive *A. phagocytophilum* samples (4/15) were submitted for sequence analysis. The sample collected in 2015 (530 bp) and one of the samples from the year 2011 revealed 100% similarity with a sequence of the 16S RNA gene of *A. phagocytophilum* isolated from mouflon blood samples in the Czech Republic (GenBank accession number EU839851). The two remaining samples showed 100% similarity to *A. phagocytophilum* isolated from sika deer spleen samples in Germany (GenBank accession number KU705182).

### Co-infections

A total of 71 co-infections were found between all the tested samples (10.2%) (Table 3). Five among these co-infections were tri-infections by *B. burgdorferi* s.l with *Rickettsia* spp. and Anaplasmataceae, and one was a tri-infection by *B. burgdorferi* s.l. and SFG *Rickettsia* and Anaplasmataceae.

**Table 3:** The observed frequency of co-infections detected by PCR assays in *I. ricinus* ticks.

	*B. burgdorferi* s.l.	*Babesia* spp.	*Rickettsia* spp.	SFG *Rickettsia*	Anaplasmataceae	*A. phagocytophilum*
*B. burgdorferi* s.l.	–	–	–	–	–	–
*Babesia* spp.	0	–	–	–	–	–
*Rickettsia* spp.	17	1	–	–	–	–
SFG *Rickettsia*	1	0	–	–	–	–
Anaplasmataceae	14	0	38	6	–	–
*A. phagocytophilum*	2	0	2	0	–	–

## Discussion

A survey for the detection of tick-borne pathogens circulating in the Pic de Bazès was conducted, following the observation of relatively low and variable kid spring survival in the local population of Pyrenean chamois [42], high infestations by ticks on some animals, and the presence of three species of tick-borne *Babesia* [13]. In this study, *B. burgdorferi* s.l., *Babesia* spp., *Rickettsia* spp. and *Anaplasma phagocytophilum* were detected in *I. ricinus* ticks collected in 2009, 2011, 2013 and 2015.

All collected ticks were identified as *I. ricinus*, which is considered as one of the most commonly found tick species in Europe [6]. In this study, the overall number of adult ticks (*n* = 196) was considerably lower than the number of nymphs collected (*n* = 1775), as in many other studies using the similar dragging technique for collecting questing ticks [3, 38]. This confirms the low performance of this technique to collect adult ticks [38]. Tick abundance also varied greatly according to the year of collection which can be explained by yearly variations in environmental factors known to affect the abundance and activity of ticks, such as relative humidity, temperature, soil type, vegetation and animal density [21], and also by a lower number of sampling sites in 2015 compared to previous years (8 vs. 15).

The overall prevalence of *B. burgdorferi* s.l. in our study was found to be close to that found in a similar study carried out in Italy (5.8%) [38], but lower than that reported in Switzerland (20–35%) [20]. In this study, except in 2011, the overall infection prevalence of nymphs appeared to be significantly higher than that of adults, while the opposite was expected. In fact, this spirochaete is known to be acquired by ticks *via* blood feeding and interstadial transmission, and therefore adults are expected to show a higher prevalence than nymphs as observed in *I. ricinus* in Switzerland [20]. However, such findings are not always consistent, since other similar studies found no significant difference in relevance to tick stage [30].

Sequence analysis of positive samples collected in 2011 and 2013 showed 99–100% identity to multiple species of the *B. burgdorferi* s.l complex, such as *B. burgdorferi* s.s. and *B. garinii*. As a consequence, sequencing of these samples does not allow the deduction of the identified pathogen species. On the other hand, positive samples collected in 2009 showed 100% identity with *B. afzelii*. While *B. afzelii* and *B. garinii* are mainly associated with rodents and birds [26], they are both responsible for clinical disease in humans. In fact, in the administrative region of Occitanie, the incidence rate of human Lyme borreliosis was found to be between 20 and 59 per 100,000 inhabitants in a study conducted from 2009 to 2012 [46]. Therefore, the identification of pathogenic *B. burgdorferi* s.l. in ticks of the Pic de Bazès should raise awareness of the possibility of contracting Lyme borreliosis in this area [20].

In our study, *Babesia* spp. prevalence was 0.4% which is in accordance with similar studies carried in European countries such as Poland, Germany, Slovakia and Switzerland where infection rates were between 0.4 and 4.5% [16, 44]. In fact, *I. ricinus* carrier rates of *Babesia* spp. rarely exceed this range [30].

All three positive samples were sequenced and showed over 99% similarity with *Babesia* sp. EU1, which is in accordance with its detection from six Pyrenean chamois of the Pic de Bazès in 2008 [13]. These animals were in apparent healthy condition but *Babesia* sp. EU1 is considered to be one of the *Babesia* species causing clinical disease in humans and also in chamois [13, 30]. However, in the latter report, *B. capreoli* and *B. divergens* were also detected [13], which was not the case in the current study. In another study conducted in Switzerland, *Babesia* sp. EU1 was identified in 64.3% of positive *Babesia* spp. samples while *B. divergens* was identified in 17.9% [25], which highlights the importance of *Babesia* sp. EU1 in relation to other *Babesia* species. Therefore, the absence of *B. capreoli* and *B. divergens* detection in this study could be simply explained by the low *Babesia* spp. prevalence. Nevertheless, these findings prove the circulation of *Babesia* spp. in ticks of the Pic de Bazès, which might influence the demography of the local population of Pyrenean chamois [13].

In the current study, overall *Rickettsia* spp. prevalence was 17.6% with the highest prevalence being found in 2011 (38.5%) and the lowest in 2009 (0.6%). A study on the detection of *Rickettsia* spp. in *I. ricinus* collected in 2007 from Pyrenean chamois in the same area as this study revealed a prevalence of 6% [6]. In Germany, the detection of *Rickettsia* spp. in questing *I. ricinus* also revealed a large variation between 2005 and 2015 with prevalence ranging from 26.2 to 50.8% [3].

We had significantly higher infection rates in adult ticks compared to nymphs, as previously observed [3]. However, the effect of tick stage on prevalence of *Rickettsia* spp. varies between studies with some revealing the absence of effect, which is often assumed to result from the dual interstadial and transovarian transmission of this pathogen [15].

SFG *Rickettsia* was detected in 8.1% of positive *Rickettsia* spp. samples. However, it is important to note that this is not the real SFG *Rickettsia* prevalence, since an additional PCR assay amplifying the ompB gene is necessary to confirm that negative samples are indeed non-SFG *Rickettsia* [10]. Therefore, *Rickettsia* spp. positive samples not exhibiting the ompA gene should not systematically be considered as symbiotic bacteria. This justifies the submission of such samples for sequence analyses.

Sequence analyses revealed 100% identity with *R. helvetica* and *R. monacensis*. The former is considered the predominant *Rickettsia* species in Germany, while the latter is associated with sporadic cases [3]. This is, to our knowledge, the first identification of *R. monacensis* in France. This bacterium has been detected in *I. ricinus* in different countries in Europe, with prevalence in ticks ranging from 1 to 57%, depending of the studies. It has also been detected in lizard tissues, but more importantly, was identified as a human pathogen in two patients in Spain [35].

*R. helvetica* was similarly detected in ticks collected from Pyrenean chamois from the Pic de Bazès [6], with 98.9% identity. In addition, our study has detected similarity with *R. monacensis*, an emerging human pathogen which was not found in the previous analyses of ticks from the Pic de Bazès [6].

*A. phagocytophilum* is an obligate intracellular bacterium of the Anaplasmataceae family, infecting neutrophils. In ruminants, *A. phagocytophilum* causes Tick-Borne Fever, a mild disease with non-specific signs (fever, lethargy and anorexia). This bacterium is maintained by animals recovering from clinical disease that become persistent carriers [8], while in wild ecosystems, it is maintained through enzootic cycles between ticks and wild and domestic animals (e.g., cervids, rodents, migratory birds, sheep) acting as reservoirs [34, 48].

*A. phagocytophilum* is mainly transmitted in Europe by *I. ricinus* ticks [23]. Prevalence of the pathogen in Europe ranges from 0.4 to 67%, depending on the studied area. In fact, prevalence in questing *I. ricinus* ranged between 1.0 and 17.4% in Germany; 3.7 and 20.5% in Spain; and 1.5 and 24.4% in Italy [45]. In this study, *A. phagocytophilum* was detected in 6.1% of the tested *I. ricinus.* Studies conducted in different areas of France on questing ticks are in accordance with our results with prevalence not exceeding 10.7% [1, 15, 40]. Ticks tested in our study were collected from an area with vegetation mainly represented by pastures and shrubs, surrounded by woodland. Forest fragmentation is considered to favor the prevalence of *A. phagocytophilum* in woodland and pastures due to the abundance of rodents in this type of environment [15]. Therefore, the Pic de Bazès is potentially a favorable zone for the maintenance of this bacterium, which could be a threat to humans visiting the site.

The largest number of co-infections found in this study were between Anaplasmataceae and *Rickettsia* spp. (*n* = 38) and between *Borrelia burgdorferi* s.l. and *Rickettsia* spp. (*n* = 17). While *Rickettsia* spp. detected in this study contains a large number of potentially pathogenic bacteria, Anaplasmataceae contain only a few, with the remainder being symbiotic bacteria. As a result, co-infections with Anaplasmataceae should be interpreted carefully in order to avoid overestimating the importance of these bacteria as pathogens. Since *B. burgdorferi* s.l., *Rickettsia* spp. and Anaplasmataceae share similar reservoir hosts (i.e., rodents) as well as vector species (i.e., *I. ricinus*), significant rates of co-infection are to be expected. In fact, other studies have previously reported *A. phagocytophilum* and *Rickettsia* spp. co-infections [3].

## Conclusion

Following the detection of *Babesia* sp. EU1 in Pyrenean chamois of the Pic de Bazès, we also detected the same pathogen in questing ticks in our study. In addition, we detected *B. burgdorferi* s.l., *Babesia* spp., *A. phagocytophilum* and SFG *Rickettsia*, with sequence analysis revealing, to our knowledge, the presence of *Rickettsia monacensis* for the first time in France. Furthermore, the zoonotic nature of these tick-borne infections indicates the need to raise awareness of the risk of contracting these pathogens for humans visiting the area.
